# Histone Modifications Drive Aberrant Notch3 Expression/Activity and Growth in T-ALL

**DOI:** 10.3389/fonc.2019.00198

**Published:** 2019-04-03

**Authors:** Luca Tottone, Nadezda Zhdanovskaya, Álvaro Carmona Pestaña, Michele Zampieri, Fabrizio Simeoni, Sara Lazzari, Valeria Ruocco, Maria Pelullo, Paola Caiafa, Maria Pia Felli, Saula Checquolo, Diana Bellavia, Claudio Talora, Isabella Screpanti, Rocco Palermo

**Affiliations:** ^1^Department of Molecular Medicine, Sapienza University of Rome, Rome, Italy; ^2^Department of Cellular Biotechnologies and Hematology, Sapienza University of Rome, Rome, Italy; ^3^Center for Life Nano Science@Sapienza, Istituto Italiano di Tecnologia, Rome, Italy; ^4^Department of Experimental Medicine, Sapienza University of Rome, Rome, Italy; ^5^Department of Medico-Surgical Sciences and Biotechnology, Sapienza University of Rome, Latina, Italy

**Keywords:** Notch signaling, T-cell lymphoblastic leukemia, epigenetics, JMJD3, p300, gene regulation, GSKJ4, A-485

## Abstract

T-cell acute lymphoblastic leukemia (T-ALL) is an aggressive blood cancer caused by the deregulation of key T-cell developmental pathways, including Notch signaling. Aberrant Notch signaling in T-ALL occurs by *NOTCH1* gain-of-function mutations and by *NOTCH3* overexpression. Although *NOTCH3* is assumed as a Notch1 target, machinery driving its transcription in T-ALL is undefined in leukemia subsets lacking Notch1 activation. Here, we found that the binding of the intracellular Notch3 domain, as well as of the activated Notch1 fragment, to the *NOTCH3* gene locus led to the recruitment of the H3K27 modifiers JMJD3 and p300, and it was required to preserve transcriptional permissive/active H3K27 marks and to sustain *NOTCH3* gene expression levels. Consistently, pharmacological inhibition of JMJD3 by GSKJ4 treatment or of p300 by A-485 decreased the levels of expression of *NOTCH3, NOTCH1* and of the Notch target genes *DELTEX1* and c-Myc and abrogated cell viability in both Notch1- and Notch3-dependent T-cell contexts. Notably, re-introduction of exogenous Notch1, Notch3 as well as c-Myc partially rescued cells from anti-growth effects induced by either treatment. Overall our findings indicate JMJD3 and p300 as general Notch1 and Notch3 signaling co-activators in T-ALL and suggest further investigation on the potential therapeutic anti-leukemic efficacy of their enzymatic inhibition in Notch/c-Myc axis-related cancers and diseases.

## Introduction

T-cell acute lymphoblastic leukemia (T-ALL) is an aggressive hematological malignancy caused by abnormal activity of signaling pathways regulating key stages of intrathymic lymphopoiesis. Notch signaling is an evolutionary conserved developmental pathway based on the interaction between different Notch receptors (Notch1-4) and specific ligands. The binding of a ligand allows two sequential proteolytic cleavages of the Notch receptor, mediated by ADAM metalloproteases and gamma-secretase complex, which release the intracellular active domain of Notch (NICD) from the cell membrane. Soluble NICD translocates to the nuclear compartment where it assembles a multifactorial complex to elicit its transcriptional activity (1). Among the members of Notch receptors family, proper functioning of Notch1 and Notch3 signaling is critical during physiological T cell-lineage intra-thymic development (2), and their aberrant activities perturb thymocyte differentiation and drive T-ALL pathogenesis by promoting a distinct oncogenic transcriptional program (3–10).

Notch1 signaling hyper-activation is sustained in about 60% of T-ALL patients by gain-of-function mutations occurring in *NOTCH1* gene, which promote increased stability and ligand-independent release of the N1ICD (4). Notch3 receptor has been found overexpressed in most of the patients analyzed (3), and in primary samples, unlike Notch1, its activation was preferentially associated with high expression of full-length receptor rather than with gene mutations or rearrangements (9). These findings are in line with evidence demonstrating that Notch3 receptor is more susceptible than Notch1 to spontaneous basal transcriptional activity due to ligand-independent proteolysis, even though both receptors elicit comparable levels of ligand-dependent activities (11). Overall, these observations indicate machinery regulating *NOTCH3* over-expression among the major causes of its oncogenic malfunction in this malignancy. However, molecular mechanisms sustaining *NOTCH3* expression remain mostly undefined and, although it is assumed that *NOTCH3* is a target gene of Notch1, to date it has not been clarified how its oncogenic expression/activation results to be aberrant even in T-ALL cases lacking Notch1 activation.

Notably, recent studies indicated epigenetic modifications at *NOTCH3* gene locus to drive its expression in leukemia, as it has been demonstrated to be hypermethylated in B-ALL samples not expressing *NOTCH3*, while it has been found poorly or unmethylated in T-ALL context (12). Besides, we previously demonstrated that the binding of BORIS/CTCFL at *NOTCH3* proximal promoter is critical to maintain active histone H3 tri-methylated lysine 4 chromatin mark (H3K4me3) (13). Other studies indicated the intron1 of *NOTCH3* as an enhancer region devoid of repressive H3 tri-methylated lysine 27 mark (H3K27me3) and associated with the active chromatin mark histone H3 acetylated lysine 27 (H3K27ac) in T-ALL cells, This gene region appears to be required for Notch1-dependent transcriptional activation of *NOTCH3* (14, 15).

Levels of H3K27me3 mark at gene loci result from the balance between the methyltransferase activity of the Polycomb-Repressive Complex 2 (PRC2) component EZH2 (16) and of the enzymatic activity of the H3K27 demethylases JMJD3 (also referred to as KDM6B) and UTX (also referred to as KDM6A) (17). Recently, H3K27me3 modifiers have been linked to T-ALL onset and progression and have been demonstrated to be involved in transcriptional crosstalk with Notch1. Indeed, about 25% of T-ALL patients harbor loss-of-function-mutation or deletion of *EZH2*, or of its cofactor *SUZ12* (18). Consistently, EZH2 acts as a tumor suppressor in T-ALL by antagonizing Notch signaling transcriptional activity (18). Similarly, inactivating gene lesions of *UTX* characterize a group of T-ALL patients and it has been shown that deletion of *UTX* accelerates leukemia growth in Notch1-dependent mice models (19, 20). Nevertheless, a more recent study proposed that UTX might act as a proto-oncogene in distinct subgroups of T-ALL characterized by the expression of the oncogenic transcription factor TAL1 (21). On the other hand, the H3K27 de-methylase JMJD3 has been found overexpressed in T-ALL samples when compared with physiological T-cell subsets, and it has been shown to sustain Notch1 oncogenic transcriptional program in murine models of T-ALL (19). In general, levels of H3K27ac mainly result from the balance between the enzymatic activity of the acetyltransferase p300 and of the Nucleosome Remodeling Deacetylase complex (NURD) subunits HDAC1 and HDAC2 (22). It is well accepted that p300 acts as a Notch1 transcriptional co-activator (23, 24).

Here, we further explored the interplay between Notch signaling and the above-mentioned chromatin modifiers to gain further insights into molecular mechanisms driving aberrant expression and activation of Notch3 receptor in T-ALL even in contexts lacking Notch1 activation, with the aim to reveal novel potential therapeutic targets relevant in this hematological cancer.

## Materials and Methods

### Cell Lines and Treatments

MOLT3, DND41, KOPTK1, P12/Ichikawa and TALL-1 cells were maintained in RPMI-1640 (31870025; Gibco, Carlsbad, CA, USA) containing 10% fetal bovine serum (FBS) (10270106; Gibco). HEK cells and HEK cells stably expressing full-length human Jagged1 were cultured in D-MEM (11960044; Gibco) containing 10% FBS (10270106; Gibco). To inhibit Notch S3 cleavage, MOLT3 and TALL-1 cells were treated with 10 μM gamma-secretase-inhibitor IX (DAPT) (565770; Calbiochem, Darmstadt, Germany) or with vehicle (DMSO) (D8418; Sigma-Aldrich, St Louis, MO, USA) for 48 h. For DAPT wash-out experiment, after 48 h of 10 μM DAPT treatments, cells were washed twice and cultured for 6 h in fresh medium without DAPT and in presence of 20 μg/ml of Cycloheximide (C4859; Sigma-Aldrich) (post DAPT). To block Notch signaling, P12/Ichikawa cells were cultured in medium containing 10 μg/ml of blocking anti-human Notch1 clone MHN1-519 (352104; Biolegend, San Diego, CA, USA) for 48 h, as previously described (25). Mouse IgG1 (40140; Biolegend) was used as isotype control.

To trigger Notch1 signaling, 6 × 10^6^ TALL-1 cells were co-cultured on a monolayer of Jagged1-expressing HEK cells (H-J1) or HEK cells as control (H) for 48 h. Suspension cells were recovered, washed in phosphate-buffered saline (PBS) and subjected to further analysis. To inhibit enzymatic activity of JMJD3 or of p300, cells were exposed to 2 μM GSK J4 HCL (GSKJ4) (S7070; Selleckchem, Houston, TX, USA) or to 5 μM A-485 (HY-107455; MedChemExpress, Monmouth Junction, NJ, USA**)** for times indicated in figures.

### Protein and Histones Extracts Preparation, Western Blot, and Antibodies

To obtain total protein extracts, cells were lysed in Laemmli buffer (2X Laemmli Sample Buffer (1610737; Biorad, Hercules, CA) by sonication and clarified at 10.000 × g for 10 min. Before immunoblotting, samples were added with β-mercaptoethanol (M6250; Sigma-Aldrich) and boiled for 10 min. To analyze histone mark levels, histones were extracted by using the acid extraction protocol by Abcam (Cambridge, UK). For immunoblotting, protein and histone extracts were run on SDS-polyacrylamide gels and transferred to nitrocellulose membranes (1620115; Biorad). Blots were incubated with antibodies against: Notch1Val1744 (4147; Cell Signaling Technology, Beverly, MA, USA), Notch1 D1E11 (3608; Cell Signaling Tecnology), Notch3 (2889; Cell Signaling Technology), H3K27me3 (9733; Cell Signaling Technology), H3K27ac (4353; Cell Signaling Technology), H3 (07-690; Merck Millipore, Darmstadt, Germany), PARP (9542; Cell Signaling Technology), p27 (3688; Cell Signaling Technology), β-actin (A5441; Sigma-Aldrich), Myc Tag (06-549; Sigma-Aldrich), HA (16B12; Covance Inc, Princeton, NJ, USA) followed by hybridization with Antibodies HRP conjugated: anti-rabbit (A120-108P; Bethyl Laboratories, TX, USA) or anti-mouse (A90-116P; Bethyl Laboratories).

For Chip experiments following antibodies were used: Notch3 (2889; Cell Signaling Technology), Notch1 (3608; Cell Signaling Technology), H3K27me3 (07-473; Merck Millipore, Darmstadt, Germany), H3K27ac (4353; Cell Signaling Technology), H3K4me3 (07-473; Merck Millipore), JMJD3 (ab38113; Abcam, Cambridge, UK), p300 (sc-584, Santa Cruz Biotechnology, Santa Cruz, CA, USA), normal rabbit IgG (sc-2027; Santa Cruz Biotechnology) and normal mouse IgG (sc-2025; Santa Cruz Biotechnology).

### RT-PCR Analysis

Total RNA was isolated by using TRIZOL reagent (15596018; Invitrogen, Carlsbad, CA, USA) as described previously (26). cDNA was synthetized by using High Capacity cDNA Reverse Transcription Kit (4368814; Applied Biosystems, Foster City, CA, USA) according to the manufacturer's protocol. Taqman Gene Expression Master Mix (4304437) and Taqman Gene Expression Assays for *NOTCH1* (Hs01062014_m1), *NOTCH3* (Hs00166432_m1), *DELTEX1* (Hs01092201_m1), *GAPDH* (Hs02758991_g1), and 18S (4352930E) were purchased from Applied Biosystems. Relative quantification was carried out using the comparative ΔΔCT method, as previously described (27). GAPDH or 18S were used to normalize mRNA levels. Measurements were performed in technical triplicates and figures show the averages ± SEM of at least 3 biological replicates.

### Chromatin Immunoprecipitation (ChIP)

To crosslink protein/DNA complex, cells were incubated for 10 min at 37°C after the addition of 1% formaldehyde (47608; Sigma-Aldrich) to the media. Crosslinking was quenched by the addition of 0.125 M glycine (50046; Sigma-Aldrich) to the media followed by 10 min of incubation at RT. Cells were washed twice with PBS containing protease inhibitors (S8820; Sigma-Aldrich) and then lysed in Triton Buffer (0,25% Triton X-100, 1 mM EDTA, 0,5 mM EGTA, 10 mM Tris-HCl, pH 8) containing protease inhibitors (S8820; Sigma-Aldrich) for 10 min in ice. Nuclei were lysed in Lysis Buffer (0.5% SDS, 5 mM EDTA, 50 mM Tris-HCl, pH 8) containing protease inhibitors (S8820; Sigma-Aldrich) for 10 min in ice and sonicated for 10 min (30 s on, 30 s off) by using Bioruptor (Diagenode, Liege, Belgium). Samples were centrifuged at max speed to remove debris and then diluted by adding 9 volumes of Dilution Buffer (0.01% SDS, 1% Triton X-100, 1.2 mM EDTA pH 8.0, 16.7 mM Tris-HCl pH 8.0, 167 mM NaCl) containing protease inhibitors (S8820; Sigma-Aldrich) and pre-cleared for 1 h at 4°C with 1% v/v of Protein A/G PLUS-Agarose (sc-2003, Santa Cruz Biotechnology). Ten percent of sample was then kept out as input, and the remaining sample was split into tubes and processed by immunoprecipitation. Four micrograms of each antibody or of the appropriate normal control IgG were added to each tube and incubated overnight in rotation at 4°C. Antibody–protein–DNA complexes were collected by adding to the samples 10% v/v of salmon sperm DNA/Protein A agarose (#16-157, Merck Millipore, Darmstadt, Germany) or salmon sperm DNA/Protein G agarose (16-201; Merck Millipore, Darmstadt, Germany). After 1 h of incubation, agarose pellets were washed consecutively in LSW Buffer (0.1% SDS, 1% Triton X-100, 2 mM EDTA, 10 mM Tris-HCl, pH 8, 150 mM NaCl), HSW Buffer (0.1% SDS, 1% Triton X-100, mM EDTA, 20 mM Tris-HCl, pH 8, 500 mM NaCl), LiCl Buffer (0.25 M LiCl, 1% NP-40, 1% deoxycholic acid sodium salt, 1 mM EDTA, 10 mM Tris-HCl, pH 8), and in TE Buffer (10 mM Tris-HCl pH 8.0, 1 mM EDTA). DNA-protein complexes were eluted by adding Elution Buffer (1% SDS, 10 mM Tris-HCl pH 8,5 mM EDTA) to samples, including Input samples, followed by 15 min of shaking at 1,000 RPM at 65°C. Elution procedure was repeated two time, and followed by treatment with proteinase K (P2308; Sigma-Aldrich). Protein-DNA crosslinks were reversed at 65°C overnight. DNA was extracted by Phenol/Chloroform, precipitated by using ethanol and glycogen, air-dried, and re-suspended in H2O. Relative *NOTCH3* fragment enrichments were determined by subsequent SYBR green qPCR by using the following primers previously described (14): Forward N3 INTRON1: GTCTCAGCACACCCCATTCT; Reverse N3 INTRON1: AACCACAAAGCAGGGGAAG; Forward N3 UP-TSS: TGGCCTCAGTTTCCAGAGTT; Reverse N3 UP-TSS: CACACCCAACCTCGTGAAC. PCR reactions were run at 95°C for 10 min, followed by 40 cycles at 95°C for 15 s and 60°C for 30 s and was performed using the ViiA™ 7 Real-Time PCR System detection system (Applied Biosystems, Foster City, CA, USA). Values were normalized to input DNA by using the % input method. Measurements were performed in technical triplicates and figures show the averages ± SEM of the measurements obtained from two biological replicates.

### Plasmids and Transfections

To generate a construct expressing GFP-fused constitutively active human Notch3 intracellular domain (hICN3), the human N3ICD fragment was amplified by using primers FW 5′-GTCATGGTGGCCCGG-3′ and REV 5′-CCAACACTTGCCTCTTGGG-3′, and cloned into the mammalian expression vector pcDNA^TM^3.1/CT-GFP TOPO according to the manufacturer's instructions (K4820-01, Invitrogen). The expression vector PIRVNeoSV containing the human c-Myc cDNA coding sequence (c-Myc) was kindly provided by Dr. Giuseppe Giannini (Sapienza University, Rome, Italy). pCMV3-HA vector containing the human EZH2 coding sequence (HA-EZH2) was purchased from Sino Biological (HG11337-CY; Sino Biological, Beijing, China).

Transient transfections with expression vectors or with the relative control plasmids were carried out by using Neon Transfection System (MPK5000; Invitrogen) as described by the manufacturer. Following 24 h of transfection of hICN3 plasmid and the relative control plasmid in DND41, about 40–50% of transfection efficiency was detected by fluorescence microscopy. The strength of HA-EZH2, hICN3, and c-Myc transfection was confirmed by western blotting shown in Figures 4B, 6B,C, respectively.

### Cell Viability Assay

Human T-ALL cell lines were seeded in 96-well plate at 5 × 10^5^ cells/ml and treated with 2 μM GSKJ4 or 5 μM A-485 for times indicated in figures. As control samples, cells were treated with equal volumes of the vehicle (DMSO). Cell viabilities were assayed by using the MTS-based assay CellTiter 96^®^ AQueous One Solution Cell Proliferation Assay (G3580; Promega, Madison, WI, USA), as previously described (28). Absorbances were measured at 490nm by using GloMax Multidetection System (Promega). Data were collected as units of absorbance (ABS) and normalized to cell proliferation percentage by following equation: % Cell Viability = (ABS_cells+compound_ – ABS_medium+compound_) / (ABS_cells+DMSO_ – ABS_medium+DMSO_) X 100. Measurements were performed in technical duplicates or triplicates and figures show the averages ± SEM of at least 3 biological replicates.

Trypan blue (T8154, Sigma-Aldrich) was used to count viable and dead cells.

### Rescue Assays

Growth sensitivity to treatment with 2 μM GSKJ4 or with 5 μM A-485 was compared between DND41 cells transduced with retroviral construct encoding the entire murine Notch1 intracellular fragment CMMP-ICN1-IRES-EGFP (mICN1) and the empty relative control vector CMMP-IRES-EGFP (empty) [previously described (29)], and between DND41 cells transiently transfected with hICN3 expression plasmid and the relative control plasmid, and between TALL-1 cells transiently transfected with the expression vector c-Myc and the cell counterparts transfected with the empty control vector. Trypan blue (T8154, Sigma-Aldrich) was used to count viable cells.

### Statistical Analysis

All statistical tests were carried out by using GraphPad Prism version 6.0 (GraphPad Software, San Diego California, USA). Statistical analysis of data between two groups was carried out by two-tailed Student's unpaired *t*-test. Multiple comparisons analysis was carried out by one-way ANOVA followed by Tukey's *post-hoc* test. Differences were considered significant when *P*-values < 0.05. Values significance: ^*^P < 0.05, ^**^P < 0.01, ^***^P < 0.001, ^****^P < 0.0001.

## Results

### *NOTCH3* Is a Direct Target of Both Notch1 and Notch3 Signaling

**Figure 1:**
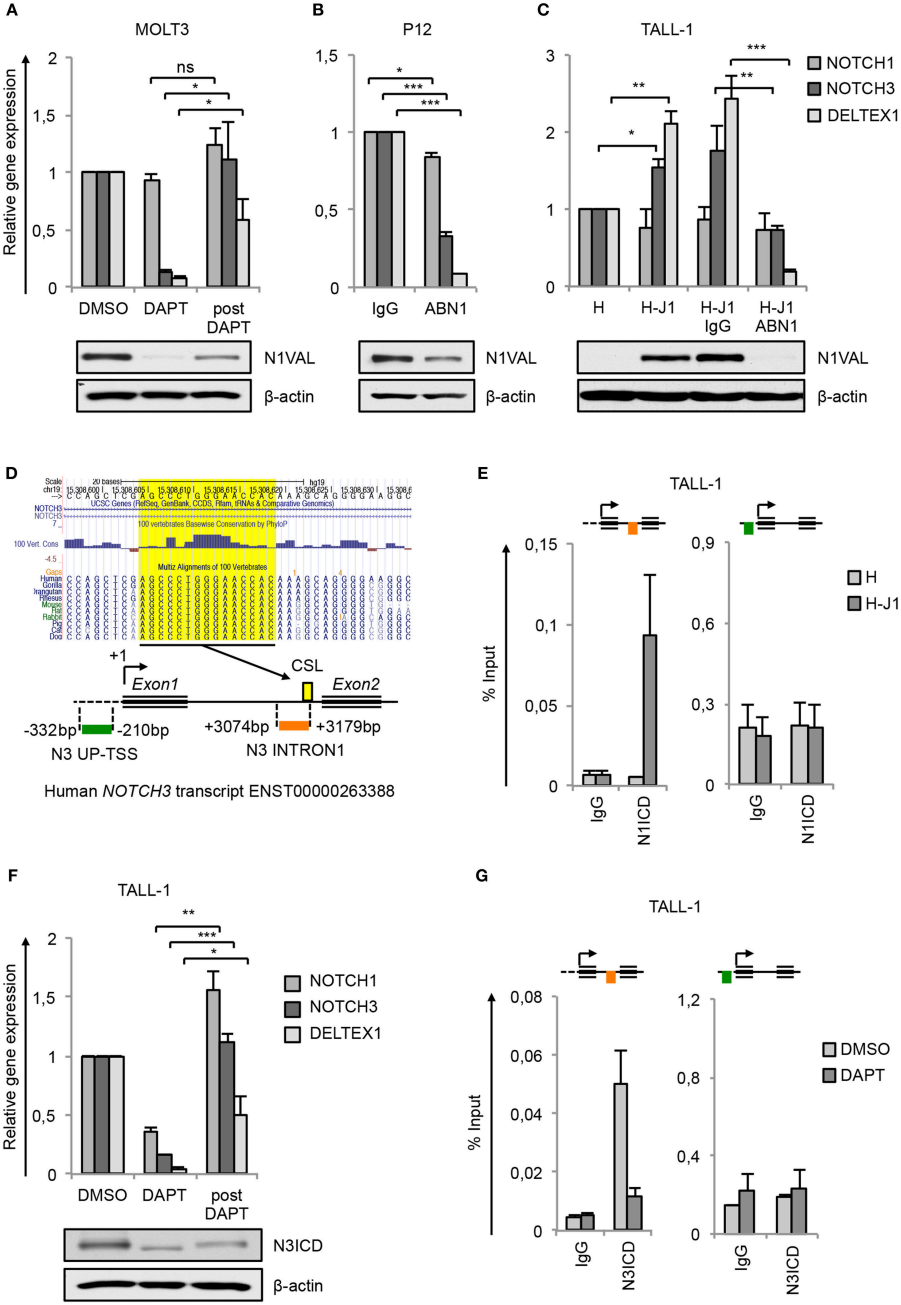
NOTCH3 is a direct target of Notch3 and Notch1 in T-ALL. Relative *NOTCH1, NOTCH3*, and *DELTEX1* gene expression (upper panels) and protein levels of endogenous Notch1 active domain (N1VAL) and β-actin (lower panels) in: **(A)** MOLT3 cells treated for 48 h with 10 μM DAPT (DAPT) or with DMSO and, after washing, incubated for further 6 h in fresh medium without DAPT and in presence of 20 μg/ml of Cycloheximide (post DAPT), **(B)** P12 T-ALL cells exposed for 48 h to anti-human blocking Notch1 antibody (ABN1) or to the isotype controls (IgG), and **(C)** TALL-1 cells co-cultured for 48 h on a monolayer of HEK cells expressing Jagged1 (H-J1) or on the not expressing Jagged1 cell counterpart (H) and treated with anti-human Notch1 blocking antibody (H-J1 ABN1) or with the isotype control (H-J1 IgG). Data represent mean values of three or four biological replicates ± Standard Error of the Mean (S.E.M.); (*n* = 3 or 4) *P < 0.05, **P < 0.01, ***P < 0.001. (**D)** UCSC Genome Browser Screenshot showing the conserved CSL/RBPjk binding site predicted by Genomatix Maltinspector at the *NOTCH3* intron1 (upper panel); a schematic representation of the human *NOTCH3* genomic region within 5′-upstream (UP-TSS) and the intron1 (INTRON1) regions showing DNA fragments amplified by PCR primers following the Chip procedures. Amplicons are indicated in green at the UP-TSS and in red at the INTRON1. Predicted TSS has been fixed as +1 bp. CSL/RBPjk (CSL) consensus site is indicated by the yellow box (lower panel). **(E)** Cross-linked chromatin derived from TALL-1 cells co-cultured for 48 h on a monolayer of H-J1 cells or on the H cell counterpart was immunoprecipitated with an antibody against the C-terminal domain of Notch1 (N1ICD) or with the isotype control (IgG) and analyzed by qPCR. The schematic position of the amplicons is shown up the charts. Data are expressed as a percentage of input and represent mean values for two biological replicates ± Standard Error of the Mean (S.E.M.); (*n* = 2). **(F)** Relative *NOTCH1, NOTCH3*, and *DELTEX1* gene expression (upper panel) and protein levels of endogenous Notch3 intracellular domain (N3ICD) and β-actin (lower panel) in TALL-1 cells treated for 48 h with 10 μM DAPT (DAPT) or with DMSO and, after washing, incubated for further 6 h in fresh medium without DAPT and in presence of 20 μg/ml of cycloheximide (post DAPT). Data represent mean values of three biological replicates ± Standard Error of the Mean (S.E.M.); (*n* = 3) *P < 0.05, **P < 0.01, ***P < 0.001. **(G)** Cross-linked protein-DNA complexes from TALL-1 cells treated with 10 μM DAPT or DMSO for 48 h were subjected to immunoprecipitation with an antibody against the C-terminal domain of Notch3 (N3ICD) or with the relative isotype control (IgG) and subjected to qPCR analysis. Schematic positions of the amplicons are shown up the charts. ChIP results are presented as the percentage of the input DNA. Data represent mean values of two biological replicates ± Standard Error of the Mean (S.E.M.); (*n* = 2). Uncropped western blots related to this figure are displayed in Supplementary Figure 1.

Previous findings indicating *NOTCH3* as a transcriptional Notch1 target gene in T-ALL (5, 6, 14, 30, 31) have been further validated here as the consequence of Notch1 signaling inhibition. Pharmacological Notch1-blockade by gamma-secretase inhibitor DAPT (DAPT) in MOLT3 cells harboring Notch1 gain-of-function mutation resulted in almost complete clearance of the active N1ICD (N1VAL) and in strong down-regulation of the mRNA levels of *NOTCH3* and of Notch target gene *DELTEX1*, that were recovered by subsequent Notch1 signaling reactivation following DAPT removal treatment (post DAPT) (Figure 1A). Likewise, the exposure to Notch1-blocking antibody (ABN1) prevented Notch1 activation and abrogated *NOTCH3* and *DELTEX1* expression in P12-ICHIKAWA cells (referred to as P12 cells) displaying constitutive Notch1 activity (Figure 1B). Notably, also *NOTCH1* gene expression was slightly affected (Figure 1B).

To investigate whether *NOTCH3* transcriptional control by Notch1 could be functionally related to specific cell contexts, we analyzed modulation of *NOTCH3* gene expression in response to Notch1 on/off assay in TALL-1 cells that express the full-length form of Notch1 receptor lacking ligand-independent signaling activation (9). For this purpose, TALL-1 cells have been co-cultured on a mono-layer of Human embryonic kidney (HEK) cells ectopically expressing the Notch-ligand Jagged1 (H-J1) in the absence or presence of ABN1. The co-culture induced Notch1 activation in TALL-1 cells that was accompanied by increased *NOTCH3* and *DELTEX1* expression when compared with the TALL-1 cell co-cultured on the wild type HEK cells (H) (Figure 1C). Conversely, the addition of Notch1-blocking antibody (H-J1 ABN1) prevented Notch1 activation and abrogated *NOTCH3* and *DELTEX1* expression (Figure 1C).

Previously reported chromatin immunoprecipitation (ChIP) assays in Notch1-dependent SupT1 and CUTLL1 human T-ALL cell lines revealed Notch1 binding to the *NOTCH3* intron1 and indicated this region as a Notch1-responsive element in *NOTCH3* gene (14, 15). Consistently, our *in-silico* analysis by Genomatix MatInspector and by UCSC Genome Browser covering 4 kb surrounding the human *NOTCH3* transcriptional start site (TSS) revealed a CSL/RBPjk binding site at 3,174 bp downstream the TSS highly conserved in primates and rodents (Figure 1D). No potential CSL/RBPjk consensus sequences have been found upstream the TSS. To further support *NOTCH3* as a direct Notch1 target and to evaluate the context-dependency of this regulation, we analyzed by CHIP assays the endogenous binding of N1ICD to the above described *NOTCH3* intron1 fragment (N3 INTRON1) and to a region proximal to the TSS (N3 UP-TSS) in TALL-1 cells following the ligand-dependent activation of the signaling (The position of PCR amplicons is described in Figure 1D, lower panel). As expected, in TALL-1 co-cultured on H-J1 cells, N1ICD was bound to N3 INTRON1, while no signal was detected in the immunoprecipitated chromatin from TALL-1 co-cultured on control HEK cells (H) (Figure 1E). In contrast, N3 UP-TSS region, not containing CSL/RBPjk, showed no amplification and served as a negative control of the Chip assays (Figure 1E). These data confirmed *NOTCH3* as a downstream Notch1 target gene. However, they still left open the question of how its high transcription could be sustained in Notch1-defective T-ALL patients and in TALL-1 cells where Notch3 is the only source of active Notch signaling (9). Interestingly, the exposure of TALL-1 cells to DAPT treatment reduced basal expression of *NOTCH3* and *DELTEX1*, which was recovered after Notch3 receptor reactivation following DAPT removal (post DAPT) (Figure 1F). Therefore, we asked whether Notch3 could sustain its own expression by directly interacting with its gene locus. Similarly to our observations on N1ICD, we detected high levels of N3ICD enrichment at the N3 INTRON1 in TALL-1 cells, whereas its binding was absent at the UP-TSS region (Figure 1G). Furthermore, N3ICD occupancy at the intronic fragment was greatly reduced by DAPT treatment (Figure 1G).

Interestingly, while no significant variation in *NOTCH1* expression was observed in the above-mentioned Notch1 on/off assays (Figures 1A,C), except for a slight downregulation following Notch1 inhibition in P12 cells (Figure 1B), modulation of Notch3 activity resulted in changes of expression levels of *NOTCH1* mRNA in TALL-1 cells (Figure 1F). This suggested that Notch1 and Notch3 could reciprocally sustain their expression in T-ALL and that exclusive Notch3 on-state could promote its own gene expression in TALL-1 cells.

### Notch3 and Notch1 Preserve Active H3K27 Modifications at *NOTCH3* Gene Locus

We and others have previously suggested that epigenetic regulation at *NOTCH3* gene locus promotes its aberrant gene expression in some cancers, including leukemia (12–14). Interestingly, it has been shown that Notch1 sustains the expression of *HES1* and *DELTEX1* by antagonizing the binding of PRC2/EZH2 complex and by recruiting JMJD3 on their regulatory regions, therefore promoting loss of H3K27me3 (18, 19). In addition to H3K27me3 modulation, it is well-documented that dynamic changes in Notch1 or Notch3 signaling activity result in the rapid variation of H3K27ac status across the enhancer regions of several Notch responsive genes (10, 15).

Therefore, we speculated that similar epigenetic machinery might cooperate with Notch signaling in coordinating *NOTCH3* transcription in T-ALL. To evaluate this hypothesis, we analyzed the effects of Notch inhibition on the enrichment of both active H3K27ac and repressive H3K27me3 epigenetic marks and on the occupancy by the related histone-modifying factors at the *NOTCH3* gene in Notch3-dependent TALL-1 and Notch1-dependent MOLT3 T-ALL cells.

**Figure 2:**
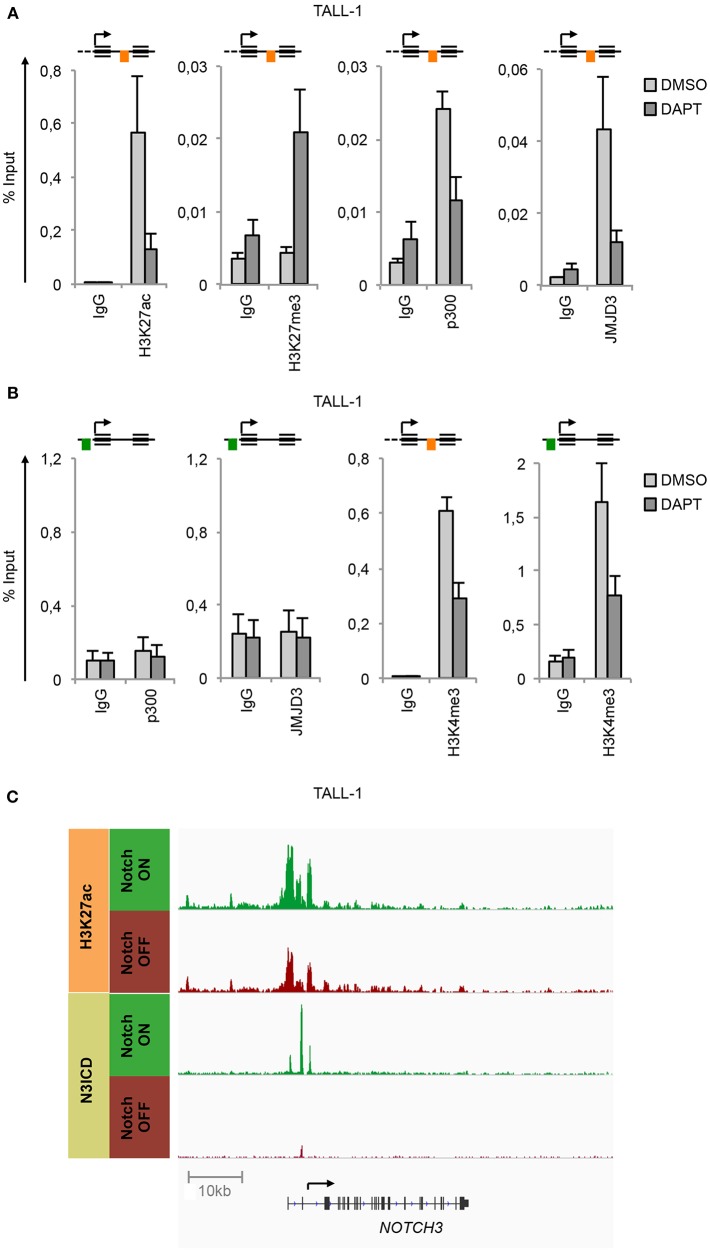
Notch3 preserves active H3K27 modification status on its gene locus. Cross-linked protein-DNA complexes from TALL-1 cells treated with 10 μM DAPT or DMSO for 48 h were subjected to immunoprecipitation with antibodies against: **(A)** H3K27ac, H3K27me3, p300, JMJD3, **(B)** p300, JMJD3, H3K4me3, and analyzed by qPCR. Immunoprecipitations against relative isotypes (IgG) were performed in parallel. Schematics position of the amplicons is shown up the charts. ChIP results are presented as the percentage of the input DNA. Data represent mean values of two biological replicates ± Standard Error of the Mean (S.E.M.); (*n* = 2). **(C)** ChIP-seq tracks of Notch3 and H3K27ac dynamic peaks visualized by IGV (32) surrounding the *NOTCH3* locus derived from previously described ChIP-seq data of human TALL-1 cells treated with gamma-secretase inhibitor (Notch OFF) or with vehicle alone (Notch ON) (GSE104262) (10).

ChIP experiments unveiled that in TALL-1 cells the binding of N3ICD at the N3 INTRON1 was strictly associated with the gain of H3K27ac and loss of H3K27me3 (Figure 2A), which was consistent with the parallel co-recruitment of the histone modifiers p300 and JMJD3 (Figure 2A). Interestingly, subsequent Notch3 inhibition by DAPT completely reversed the H3K27 epigenetic marks at the *NOTCH3* locus. Indeed, DAPT treatment resulted in H3K27me3 enrichment and H3K27ac loss and promoted the eviction of both p300 and JMJD3 from the N3 INTRON1 (Figure 2A). Of note, no binding of p300 or JMJD3 has been detected at the UP-TSS fragment (Figure 2B). In line with previous evidence showing dynamic changes in histone H3K4me3 status occurring at Notch target genes following Notch signaling modulations (33, 34), we observed high levels of H3K4me3 bound at both regulatory regions of *NOTCH3* gene in TALL-1 cells, which declined upon Notch3 blockade by DAPT treatment (Figure 2B). Recently, Choi and colleagues demonstrated that Notch1 and Notch3 drive a common oncogenic transcriptional program in T-ALL and that most of the functional Notch3 binding sites in TALL-1 cells, similarly to what was observed previously by Aster group for Notch1 in CUTLL1 cells (15), are located in enhancer regions associated with dynamic H3K27ac modulation (10). By analyzing their raw data of ChIP-seq (NCBI's Gene Expression Omnibus database; GEO GSE104262), we confirmed the presence of the above-described dynamic Notch3 binding sites located at the N3 INTRON1, which is strictly associated with change in H3K27ac marks in response to Notch3 signaling modulations in TALL-1 cells (Figure 2C).

**Figure 3:**
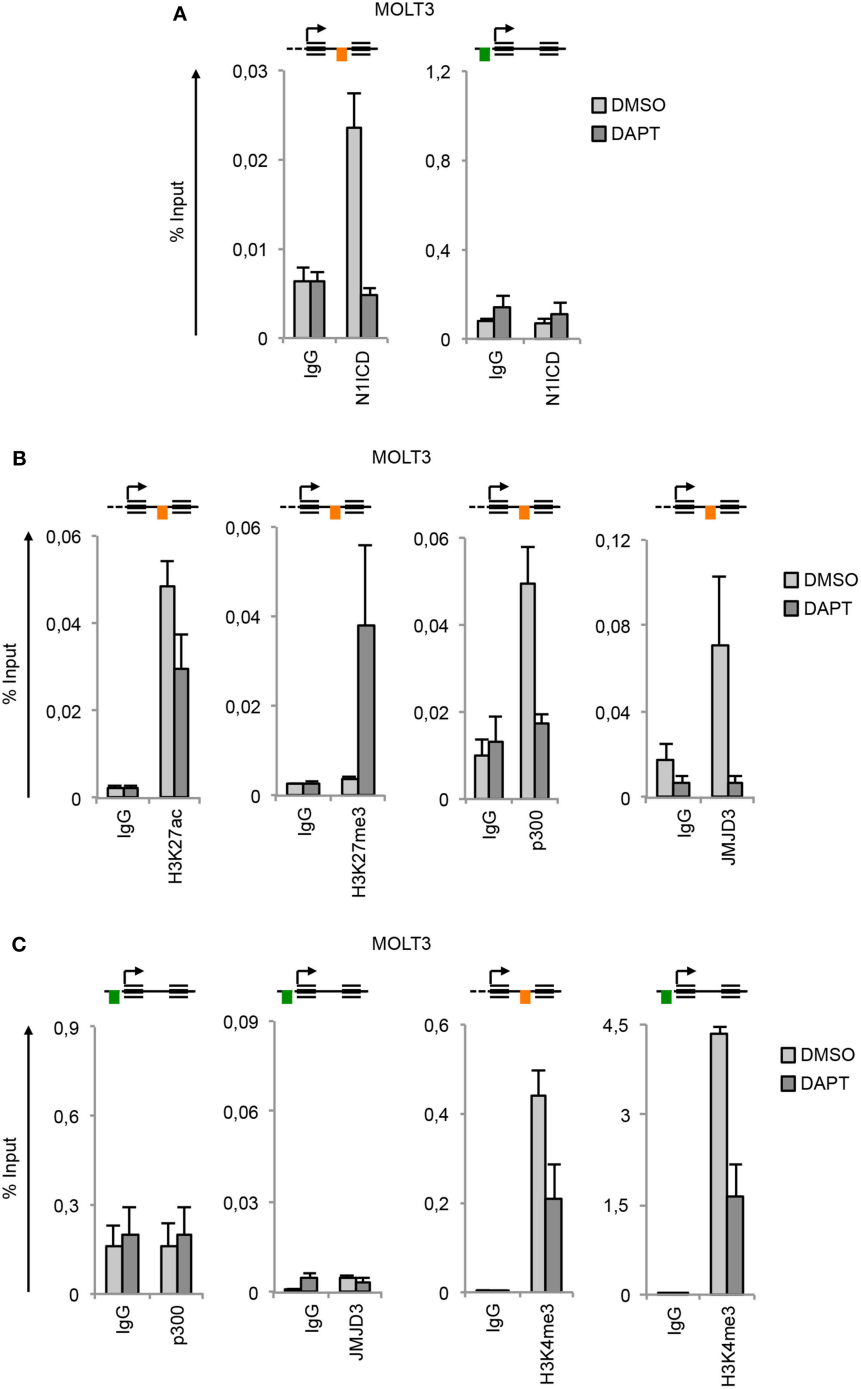
Notch1 sustains active H3K27 modification status on *NOTCH3* gene locus. Cross-linked protein-DNA complexes from MOLT3 cells treated with 10 μM DAPT or DMSO for 48 h were subjected to immunoprecipitation with antibodies against: **(A)** the C-terminal domain of Notch1 (N1ICD), **(B)** H3K27ac, H3K27me3, p300, and JMJD3, **(C)** p300, JMJD3 and H3K4me3, and analyzed by qPCR. Immunoprecipitation against relative isotypes (IgG) were performed in parallel. Schematics positions of the amplicons are shown up the charts. ChIP results are presented as the percentage of the input DNA. Data represent mean values of two biological replicates ± Standard Error of the Mean (S.E.M.); (*n* = 2).

In line with the functional similarity between the two Notches, we detected N1ICD constitutively associated to N3 INTRON1 (Figure 3A) in combination with high enrichment in H3K27ac, p300, JMJD3, and H3K4me3 in Notch1-dependent MOLT3 cells (Figures 3B,C). Similarly to TALL-1 cells, DAPT treatment of MOLT3 cells depleted N1ICD, p300 and JMJD3 interaction, dropped down H3K27ac and H3K4me3 levels (Figure 3) and increased H3K27me3 occupancy at the *NOTCH3* intronic region (Figure 3B). Again, we observed high levels of H3K4me3 at the N3 UP-TSS while JMJD3 and p300 binding at this region was detected neither in untreated nor in DAPT-treated cells (Figure 3C).

Taken together, these data indicate that Notch3 and Notch1 promote the recruitment of JMJD3 and p300 to the *NOTCH3* gene locus, which in turn preserves permissive local histone H3K27 marks along with H3K4 trimethylation to sustain gene expression.

### GSKJ4 and A-485 Impair T-ALL Cells Viability by Targeting Oncogenic Notch/c-Myc Axis

To further assess whether JMJD3 and p300 are required for *NOTCH* genes transcriptional activation and to study their functional role in Notch-dependent T-ALL cell contexts, we evaluated the effects of the inhibition of their enzymatic activities on the expression of *NOTCH3, NOTCH1* and most prominent Notch target genes and on the proliferation rate in different T-ALL cell lines. The inhibition of histone modifiers activity has been achieved by treating for 48 h cells with the commercially available specific inhibitors GSKJ4 for JMJD3 (19) or A-485 for p300/CBP (35).

**Figure 4:**
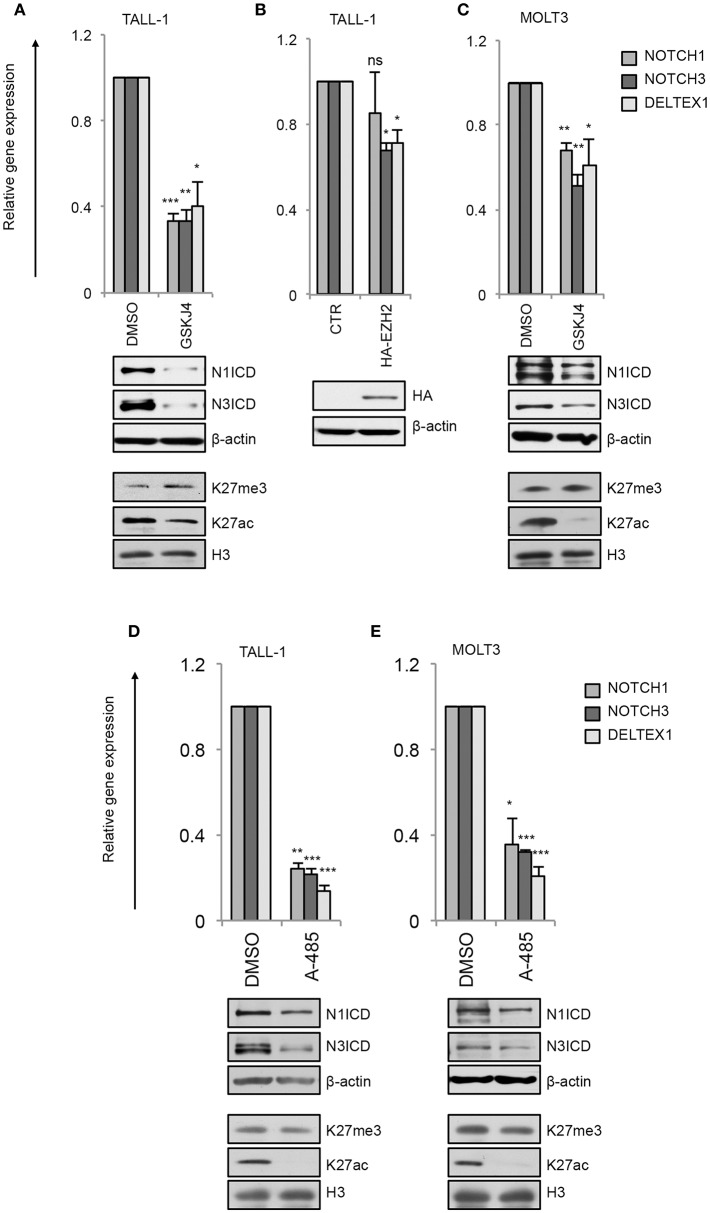
GSKJ4 and A-485 treatments modulate Notch receptors expression and activity. Relative *NOTCH1, NOTCH3*, and *DELTEX1* gene expression (upper panels) and N1ICD, N3ICD, β-actin, H3K27me3, H3K27ac, and H3 total expression levels (lower panels) in: **(A)** TALL-1 or **(C)** MOLT3 cells treated for 48 h with 2 μM GSKJ4 or with DMSO. **(B)** Relative *NOTCH1, NOTCH3*, and *DELTEX1* gene expression (upper panel) and HA and β-actin protein levels (lower panel) in TALL-1 cells transfected with HA-tagged EZH2 expression vector (HA-EZH2) or with the empty control vector. Relative *NOTCH1, NOTCH3*, and *DELTEX1* gene expression (upper panels) and N1ICD, N3ICD, β-actin, H3K27me3, H3K27ac, and H3 total expression levels (lower panels) in: **(D)** TALL-1 or **(E)** MOLT3 cells treated for 48 h with 5 μM A-485 or DMSO. Data represent mean values of three biological replicates ± Standard Error of the Mean (S.E.M.); (*n* = 3) *P < 0.05, **P < 0.01, ***P < 0.001. Uncropped western blots related to this figure are displayed in Supplementary Figures 2–4.

By this approach, we found that GSKJ4 treatment abrogated the expression of *NOTCH3* and *NOTCH1* at both gene and protein levels and reduced the transcription of Notch target gene *DELTEX1* in T-ALL cells (Figures 4A,C). Confirming the bona fide of the treatment, we found that GSKJ4 exposure was associated with increased H3K27me3 and decreased H3K27ac global accumulation (Figures 4A,C, lower panels). Previous evidence indicated that Notch1 activation antagonized the binding of EZH2 (the JMJD3 enzymatic counterpart) on target genes to allow the loss of H3K27me3 (18). Thus, we speculated that in TALL-1 cells, harboring *EZH2* missense mutations (20), *EZH2* loss of function might potentiate JMJD3 activity to preserve an open chromatin conformation at the *NOTCH3* gene locus. Supporting this hypothesis, the ectopic expression of the wild type form of EZH2 reduced the levels of *NOTCH3* and *DELTEX1*, while unexpectedly, it did not influence significantly *NOTCH1* expression in TALL-1 cells (Figure 4B).

Consistently with the above data showing that the binding of p300 to the *NOTCH3* enhancer positively combined with the amount of the active chromatin H3K27ac and with *NOTCH3* expression following Notch on/off state (Figures 2, 3), chemical inhibition of p300/CBP by A-485 decreased *NOTCH3* expression levels (Figures 4D,E). As expected, also *NOTCH1* and *DELTEX1* levels were affected by p300/CBP inhibition in either Notch3-dependent (Figure 4D) or Notch1-dependent cells (Figure 4E). Notably, as previously shown in prostate cancer cells (35), A-485 selectively suppressed global acetylation of H3K27, without any significant alteration in global H3K27 tri-methylation status in both Notch1 and Notch3 dependent T-cells (Figures 4D,E, lower panels).

**Figure 5:**
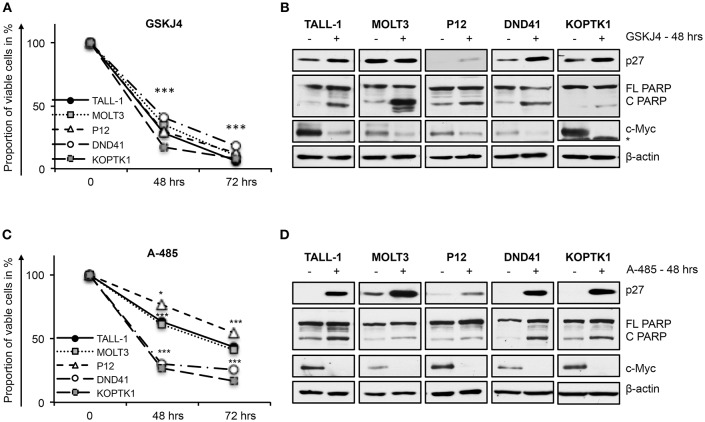
GSKJ4 and A-485 treatments inhibit viability in T-ALL cell lines. Cell viability measurement by MTS assays **(A,C)** and protein expression levels of p27, cleaved, and full-length form of PARP (C PARP and FL PARP, respectively) and c-Myc **(B,D)** in TALL-1, MOLT3, P12, DND41, and KOPTK1 cells treated for times indicated in figure with 2 μM of GSKJ4 **(A,B)** or with 5 μM of A-485 **(C,D)**. β-actin was used as loading control. Non-specific bands are indicated with asterisks. Cell viability at each time point represents the mean of three biological replicates ± Standard Error of the Mean (S.E.M.); (*n* = 3) *P < 0.05, ***P < 0.001. Uncropped western blots and additional exposures of films related to this figure are displayed in Supplementary Figures 6,7.

**Figure 6:**
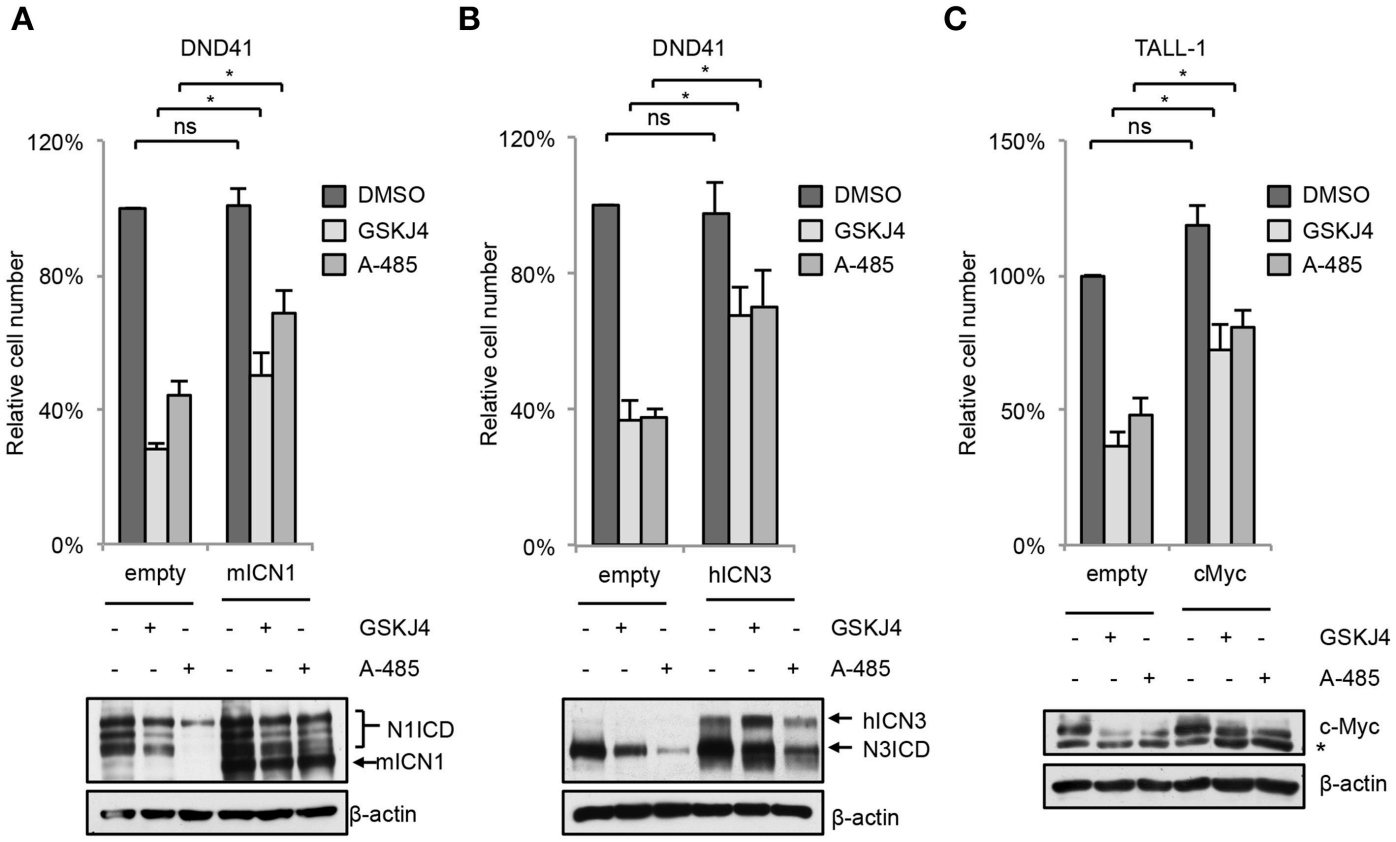
Enforced Notch/c-Myc axis partially shields from inhibitory effects of GSKJ4 and A-485 on T-ALL cell viability. Change in number of viable cells after 48 h of exposure to 2 μM GSKJ4 or to 5 μM A-485 in DND41 cells transduced with the mICN1 or with the empty control retroviruses (**A**, upper panel), in DND41 cells transiently transfected with hICN3 or with the empty vector (**B**, upper panel), and in TALL-1 cells transfected with c-Myc or with the control plasmid (**C**, upper panel). Numbers of viable cells were calculated via trypan blue exclusion assay. Relative cell number reported in charts represents the mean of at least four biological replicates normalized to cell number in DMSO-treated cells transduced or transfected with empty control ± Standard Error of the Mean (S.E.M.); *P < 0.05, **P < 0.01. Western blot analysis by using antibodies against: Notch1 in mICN1 transduced DND41 cells (**A**, lower panel), Notch3 in hICN3 transfected DND41 cells (**B**, lower panel) and c-Myc in TALL-1 cells transfected with the c-Myc expression vector (**C**, lower panel). β-actin was used as loading control. Non-specific bands are indicated with asterisks. Uncropped western blots related to this figure are displayed in Supplementary Figure 8.

In line with their anti-Notch signaling activity, treatment with low doses of either GSKJ4 or A-485 decreased the proportion of viable cells, by increasing cell death and/or decreasing the number of living cells in different T-ALL cell contexts (Figures 5A,C and Supplementary Figure 5). Consistently, both treatments reduced c-Myc expression and increased the levels of both the anti-proliferative cyclin-dependent kinase inhibitor p27^KIP1^ (p27) and the pro-apoptotic cleaved form of poly ADP-ribose polymerase PARP (Figures 5B,D). To further evaluate whether their biological effects occurred via Notch inhibition, we compared the effects of 48 h of exposure to these drugs on cell viability in DND41 cells transduced with retroviruses encoding murine N1ICD (mICN1) and with the cellular counterparts transduced with the empty retroviral vector (empty). As expected, mICN1 expression partially rescued DND41 cells from the viability inhibitory effects induced by both GSKJ4 and A-485 (Figure 6A, upper panel) and, interestingly, it preserved N1ICD endogenous expression from the effects of both drugs (Figure 6A, lower panel). Similarly, the reduction in numbers of viable cells induced by 48 h of treatments with GSKJ4 or A-485 in DND41 cells was in part prevented by the exogenous reintroduction of the human N3ICD (hICN3) (Figure 6B, upper panel) and the constitutive expression of hICN3 shielded Notch3 endogenous expression (Figure 6B, lower panel).

Since either GSKJ4 or A-485 treatment decreased c-Myc levels in T-ALL cells (Figures 5B,D), we reasoned that the axis between Notch signaling and c-Myc could be a critical target of these drugs and that c-Myc inhibition might be required for their biological effects. To evaluate this hypothesis, we compared the number of viable TALL-1 cells transiently transfected with an expression vector encoding human c-Myc with their counterpart transfected with the empty vector after 48 h of exposure to these agents. Notably, expression of exogenous c-Myc partially rescued the viability of Notch3-dependent TALL-1 cells exposed to GSKJ4 or A-485 (Figure 6C), suggesting that both treatments elicited their biological effects at least in part by targeting c-Myc signal via Notch inhibition.

## Discussion

During the last decades T-ALL cure rate has improved. However, relapse and drug-resistance represent the most common causes of treatment failure, and patients that experienced relapse undergo very poor prognosis (36–38). A deep understanding of the molecular mechanisms driving dysregulated expression and malfunction of key oncogenes or onco-suppressors in T-ALL could unveil novel potential therapeutic targets and lead to refinement of current therapies.

Given the prominent role of Notch1 and Notch3 signaling in physiological development of many tissues and in pathogenesis, chemo-resistance and relapse of different human cancers, including T-ALL, many efforts have been paid to unveil molecular mechanisms priming their activation or sustaining the strength and the activity of their signaling (1). However, less attention is paid to the upstream mechanisms regulating Notch receptors expression, particularly those involved in Notch3 transcriptional activation.

Nowadays, Notch3 is considered a direct Notch1 transcriptional target, as Notch3 expression is strictly combined with -on and -off states of Notch1 signaling in T-ALL cell lines (14, 15). Consistently, the expression of two Notches correlates during thymocytes development starting from the uncommitted CD34+CD1+ up to CD4+CD8+CD3- Double Positive (DP) stage (39). However, over than these observations, Notch1 is highly expressed in early thymocytes precursors and in mature single positive thymocytes, whereas Notch3 is preferentially highly expressed in CD4^−^CD8^−^ double negative (DN) thymocytes, particularly at the pre-TCR-dependent checkpoint stage, before shutting down in DP cells (39, 40).

In addition, high Notch3 expression and activity have been described in Notch1-lacking T-ALL patient-derived xenografts and in the established T-ALL cell line TALL-1 characterized by inactive Notch1 signaling (9).

**Figure 7:**
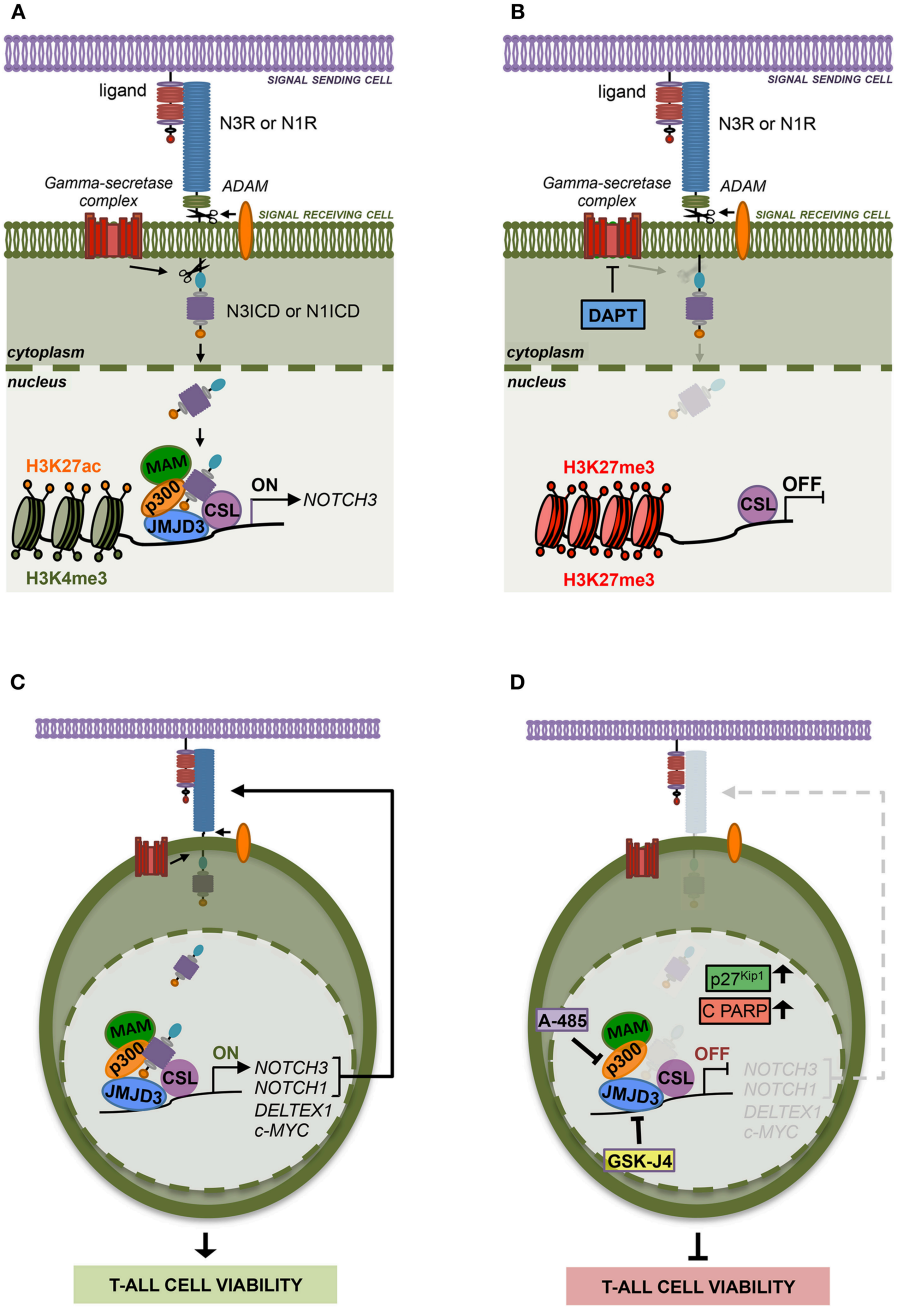
The histone modifiers JMJD3 and p300 sustain Notch signaling and cell viability in T-ALL**. (A)** Notch signaling trans-activation is primed by the binding between a Notch receptor (N3R or N1R) and a ligand expressed on the surface of two neighboring cells. Ligand interaction allows two consecutive proteolytic cleavages of the receptor by ADAM metalloproteases and by the gamma-secretase complex that releases the active Notch intracellular domain (N3ICD or N1ICD) from the cell membrane leading to its nuclear translocation. In the nucleus, N3ICD or N1ICD associates with the DNA binding protein CBF1–Suppressor of Hairless(SuH)–LAG1 (CSL) and assembles a multifactorial transcriptional complex including MAML1 complex (MAM) and p300 to promote Notch target gene expression. By our study, we demonstrated that the binding of N3ICD, as well as N1ICD, to the regulatory region of *NOTCH3*, is strictly associated with high levels of accumulation of the active chromatin marks H3K27ac and H3K4me3 on the *NOTCH3* gene locus and combines with the co-recruitment of the histone modifiers p300 and JMJD3. Moreover, we found that the interplay between Notch and these histone modifiers is required to sustain *NOTCH3* expression in T-ALL cells. **(B)** Indeed, pharmacological inhibition of the last step of Notch signaling activation, by the gamma-secretase inhibitor DAPT, restrains the binding of JMJD3 and p300 to the *NOTCH3* gene, allows H3K27me3 accumulation and counteracts *NOTCH3* expression. **(C)** Therefore, JMJD3 and p300 sustain the expression and the oncogenic transcriptional activity of Notch3 and Notch1 receptors in T-ALL cells. **(D)** Accordingly, the pharmacological inhibition of their enzymatic activity by treatment with GSKJ4 and A-485, respectively produced the following outcomes: (i) reduced *NOTCH3* and *NOTCH1* levels; (ii) decreased the expression of Notch target genes *DELTEX1* and c-MYC; (iii) promotes the accumulation of the cell cycle-regulator factors p27^Kip1^ and of the apoptosis-related cleaved form of PARP (C PARP); (ìv) impairs viability in several T-ALL cell lines.

Previous evidence suggested epigenetic machinery driving *NOTCH3* expression in different cell context. However, not much is known yet about the specific histone modifiers writing or erasing the chromatin status at *NOTCH3* gene locus especially in Notch1-lacking T-ALL subsets. Here we highlighted additional mechanistic insights into Notch3 regulation, thus advancing the understanding of distinct interplays/relationships between Notch receptors and histones modifiers relevant to Notch3 oncogenic activation in T-ALL. Supporting the previously described common transcriptional activity by Notch1 and Notch3 receptors (10), we revealed that *NOTCH3* gene expression is sustained by active Notch signaling in T-ALL cells irrespectively of the specific Notch receptor involved. We found that Notch3, as well as Notch1 signaling, activates *NOTCH3* transcription through a chromatin-modification-based mechanism by coordinating the recruitment of JMJD3 and p300/CBP chromatin remodelers, thus preserving H3K27 active epigenetic marks (Figures 7A,B). Therefore, we propose that this mechanism of transcriptional control may be a general Notch-dependent mechanism rather than a Notch1-specific one, as previously suggested, at least in some T-ALL contexts.

In addition, since Notch1 on/off states combined with changes in *NOTCH3* expression levels in Notch1-dependent T-ALL cell lines MOLT3 and P12 and given that Notch3 activity strictly correlated with *NOTCH1* expression in Notch3-dependent TALL-1 cells, we hypothesized that the reciprocal regulation by which Notch receptors modulate each other's transcriptional activity could represent a mechanism by which either Notch self-sustains a positive-feedback loop in T-ALL. Moreover, the expression of both receptors and their target genes was decreased by GSKJ4 and A-485 treatment, therefore indicating JMJD3 and p300 as major drivers of Notch receptors' transcriptional activation and activity (Figures 7C,D).

Although in the last decades several potential anti-Notch approaches have been proposed (41, 42), to date, the most explored class of Notch blocking agents, the gamma-secretase inhibitors (GSIs), is not effective in all contexts and often causes intestinal goblet cell metaplasia (43). Therefore, identification of novel key factors and mechanisms sustaining growth in T-ALL could drive the design of advanced therapeutic strategies avoiding severe GSI-related side effects. In line with this aim, we found that GSKJ4 treatment, as well as exposure to A-485, suppressed the viability of human T-ALL cell lines by promoting the accumulation of anti-proliferative factor p27 and related to apoptosis cleaved form of PARP and by suppressing protein levels of the oncogenic Notch target c-Myc (Figures 7C,D). Notably, enforced expression of the active domain of both Notch1 and Notch3 partially rescued DND41 cell lines from anti-growth effects induced by GSKJ4 and A-485, thus indicating that Notch inhibition is responsible, at least in part, of the cell viability-inhibiting effect of both drugs. Subsequent investigations in TALL-1 cells indicated that biological effects associated to GSKJ4 and A-485 might be mostly due to the inhibition of the Notch/c-Myc regulatory axis, as both treatments converged to c-Myc suppression and its enforced expression preserved cells from the treatment-associated effects on T-ALL cell viability. These last findings further indicate c-Myc as a prominent mediator of Notch oncogenic function that is consistent with the essential role of c-Myc in Notch1-driven leukemia (5, 44, 45) and with its property to rescue T-ALL cells from the anti-growth effects of Notch1 inhibition by GSI treatment (6, 44).

Overall, our study supports previous researches indicating GSKJ4 as a promising therapeutic agent in Notch1- and TAL1- dependent T-ALL (19, 21), while laying the basis for extending its potential use also to Notch3- and c-Myc- dependent T-cell leukemia contexts. Besides, it suggests further investigation on the targeting of p300/CBP by the A-485 compound as an additional therapeutic option against T-cell hematopoietic malignancies. Notably, efficacy and safety of these two epigenetic drugs is supported by previous pre-clinical studies showing that GSKJ4 and A-485 exerted anti-tumor activities in T-ALL and prostate cancer mice models, respectively, without evident adverse effects (19, 21, 35).

## Data Availability

The raw data supporting the conclusions of this manuscript will be made available by the authors upon considerable request.

## Author Contributions

LT, NZ, and ÁC performed experiments and analyzed data. FS, SL, and VR performed experiments. MZ, MP, PC, MF, SC, DB, and CT commented on the paper. IS and RP designed experiments, analyzed data and wrote the paper.

### Conflict of Interest Statement

The authors declare that the research was conducted in the absence of any commercial or financial relationships that could be construed as a potential conflict of interest.

## Abbreviations

T-ALL: T-cell acute lymphoblastic leukemia
ADAM: a disintegrin and metalloproteinase
NICD: Notch intracellular domain
N1ICD: Notch1 intracellular domain
B-ALL: B-cell Acute Lymphoblastic Leukemia; BORIS/CTCFL
H3K4me3: histone H3 lysine 4 tri-methylated
H3K27me3: histone H3 lysine 27 tri-methylated
H3K27ac: histone H3 lysine 27 acetylated
H3K27: histone H3 lysine 27
PRC2: polycomb repressive complex 2
EZH2: Enhancer Of Zeste 2 Polycomb Repressive Complex 2 Subunit
JMJD3: Jumonji Domain-Containing Protein 3
UTX: Ubiquitously-Transcribed X Chromosome Tetratricopeptide Repeat Protein
NURD: Nucleosome Remodeling and Deacetylase
HDAC1: Histone Deacetylase 1
HDAC2: Histone Deacetylase 2
GFP: Green Fluorescent Protein
N3ICD: Notch3 intracellular domain
TSS: transcriptional start site
GSI: gamma-secretase inhibitor
CSL: CBF-1/SuH/Lag-1
RBPjk: recombination signal binding protein for immunoglobulin Kappa J Region
PARP: Poly(ADP-Ribose) Polymerase.
