# Supplementary material for: Histone Modifications Drive Aberrant Notch3 Expression/Activity and Growth in T-ALL
Source: Front Oncol. 2019 Apr 3;9:198. doi: 10.3389/fonc.2019.00198 (PMC6456714; doi:10.3389/fonc.2019.00198)
Supplement: Supplementary file 1 [file Data_Sheet_1.pdf]

Supplementary Material

Histone modifications drive aberrant Notch3 expression/activity and growth in T-ALL

Luca Tottone[1,§,‡], Nadezda Zhdanovskaya[1,§, ⌘] , Álvaro Carmona Pestaña[1], Michele Zampieri[2], Fabrizio Simeoni[1,∂], Sara Lazzari[1], Valeria Ruocco[1], Maria Pelullo[3], Paola Caiafa[2], Maria Pia Felli[4], Saula Checquolo[5], Diana Bellavia[1], Claudio Talora[1], Isabella Screpanti[1,*] and Rocco Palermo[1,*].

[1]Department of Molecular Medicine, Sapienza University of Rome, Rome, Italy;
[2] Department of Cellular Biotechnologies and Hematology, Sapienza University of Rome, Rome, Italy;
[3]Center for Life Nano Science@Sapienza, Istituto Italiano di Tecnologia, Rome, Italy;
[4]Department of Experimental Medicine, Sapienza University of Rome, Rome, Italy;
[5]Department of Medico-Surgical Sciences and Biotechnology, Sapienza University of Rome, Latina, Italy.

***Correspondence:**
Rocco Palermo
rocco.palermo@uniroma1.it;
Isabella Screpanti
isabella.screpanti@uniroma1.it.

[‡]present address: Rutgers Cancer Institute of New Jersey, Rutgers University, New Brunswick, NJ, USA
[∂]present address: Cancer Research UK Manchester Institute, The University of Manchester, Manchester , UK.
[⌘]present address: Department of Physics, Sapienza University of Rome, Rome, Italy.

§These authors should be regarded as equal contributors (joint First Authors*)*.

1 Supplementary Figures

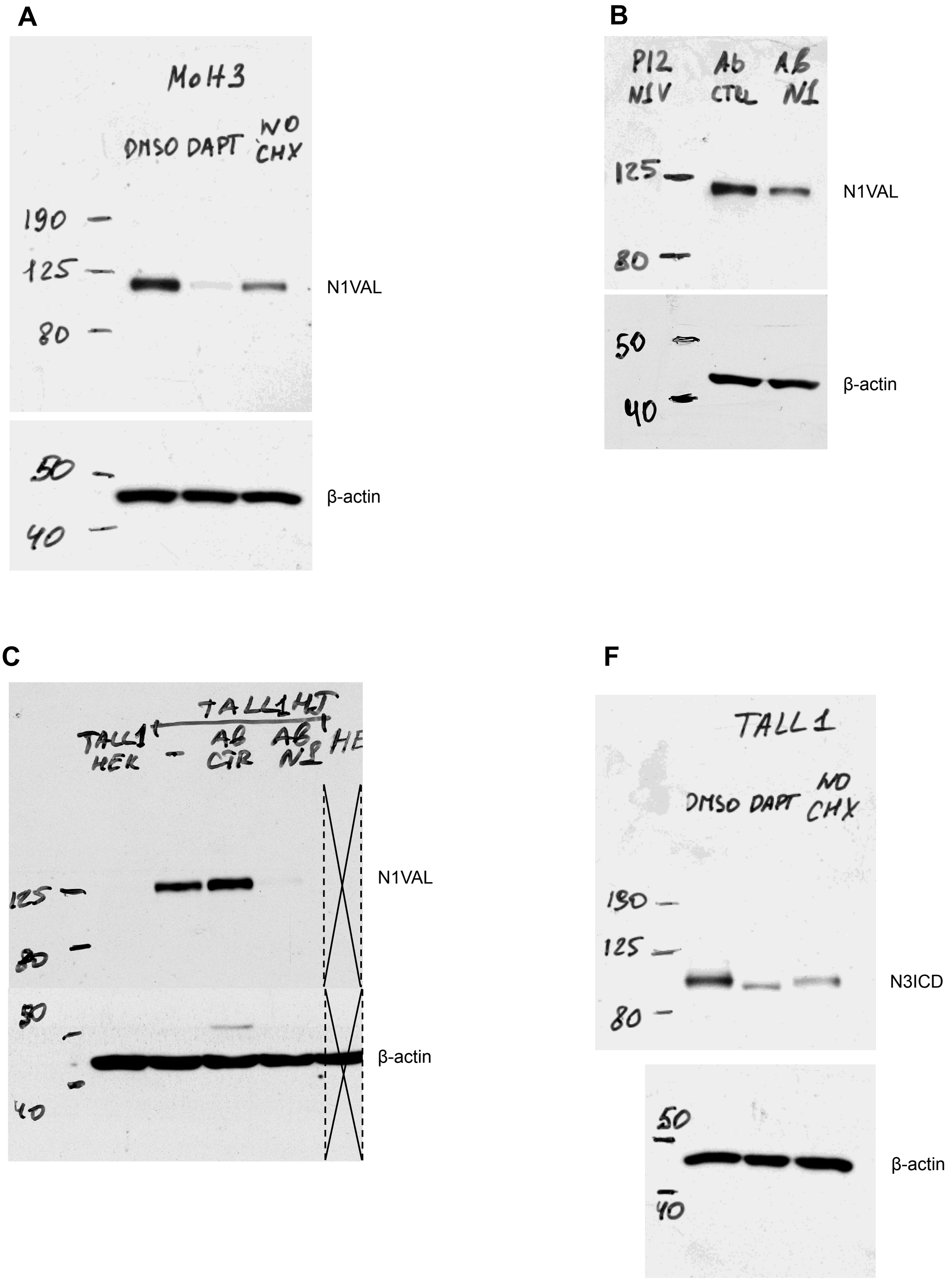

Supplementary Figure 1. Uncropped Western Blots related to main Fig. 1 A, B, C and F.

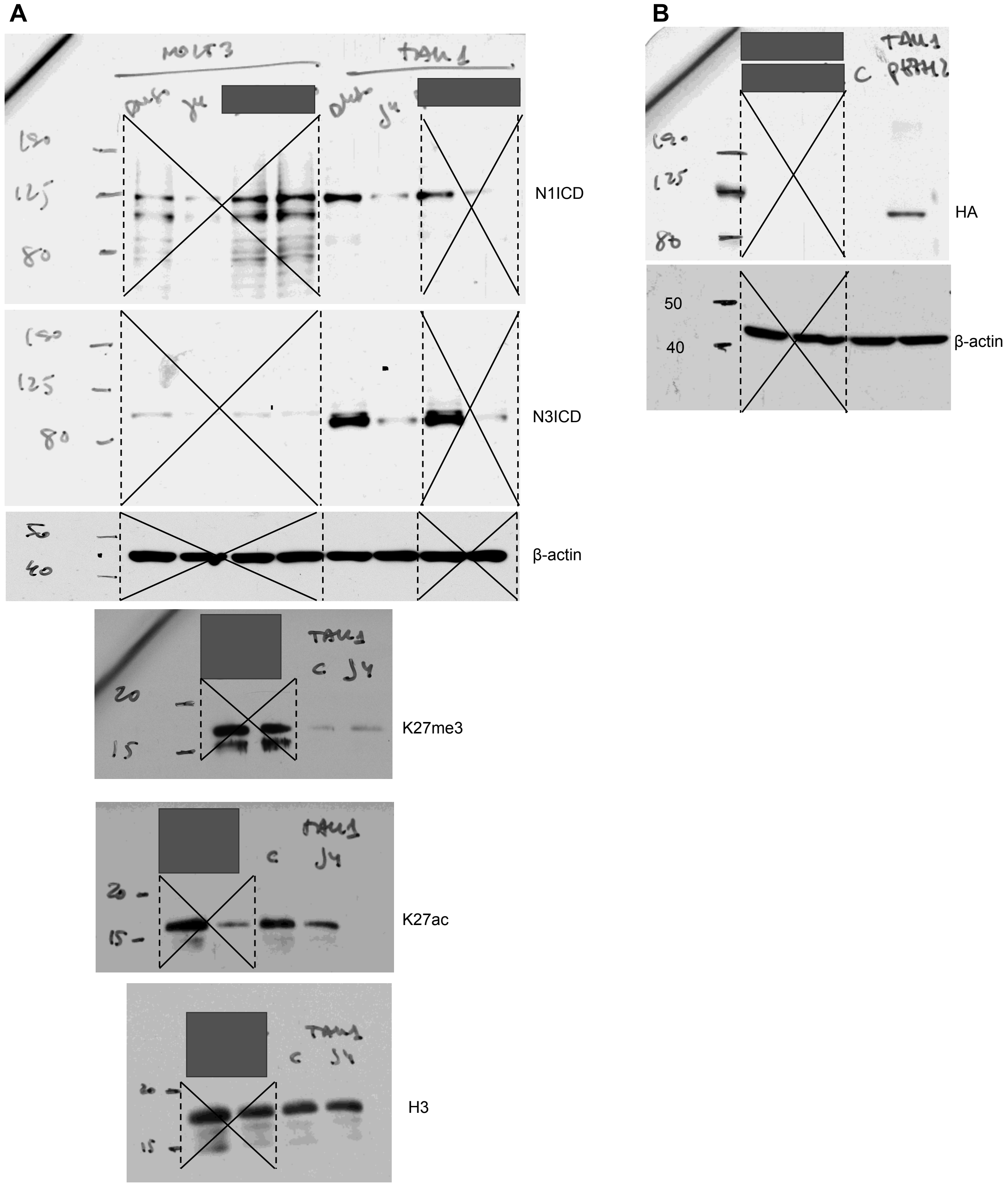

Supplementary Figure 2. Uncropped Western Blots related to main Fig. 4A and 4B.

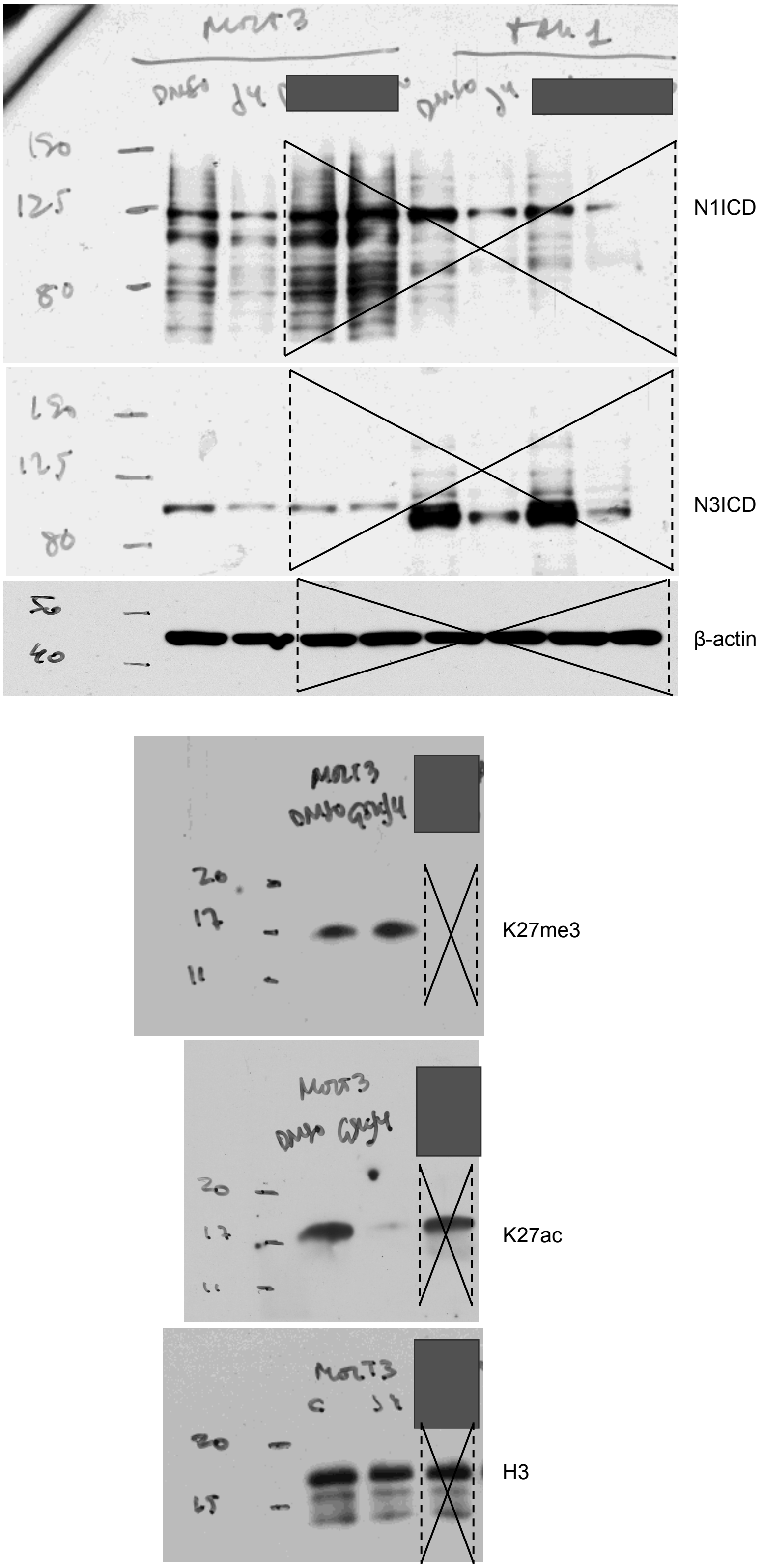

Supplementary Figure 3. Uncropped Western Blots related to main Fig.4C.

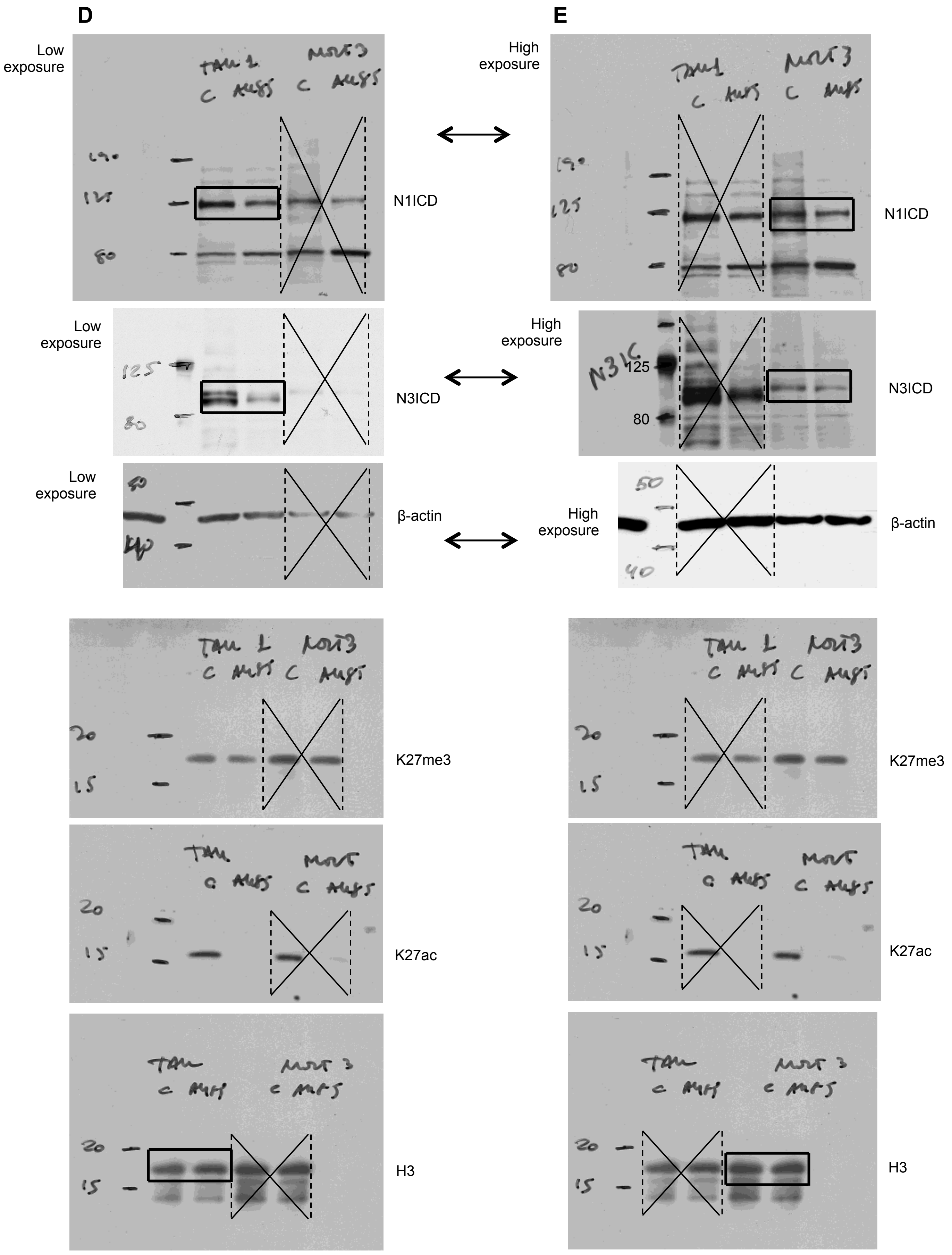

Supplementary Figure 4. Uncropped Western Blots related to main Fig. 4D and 4E.

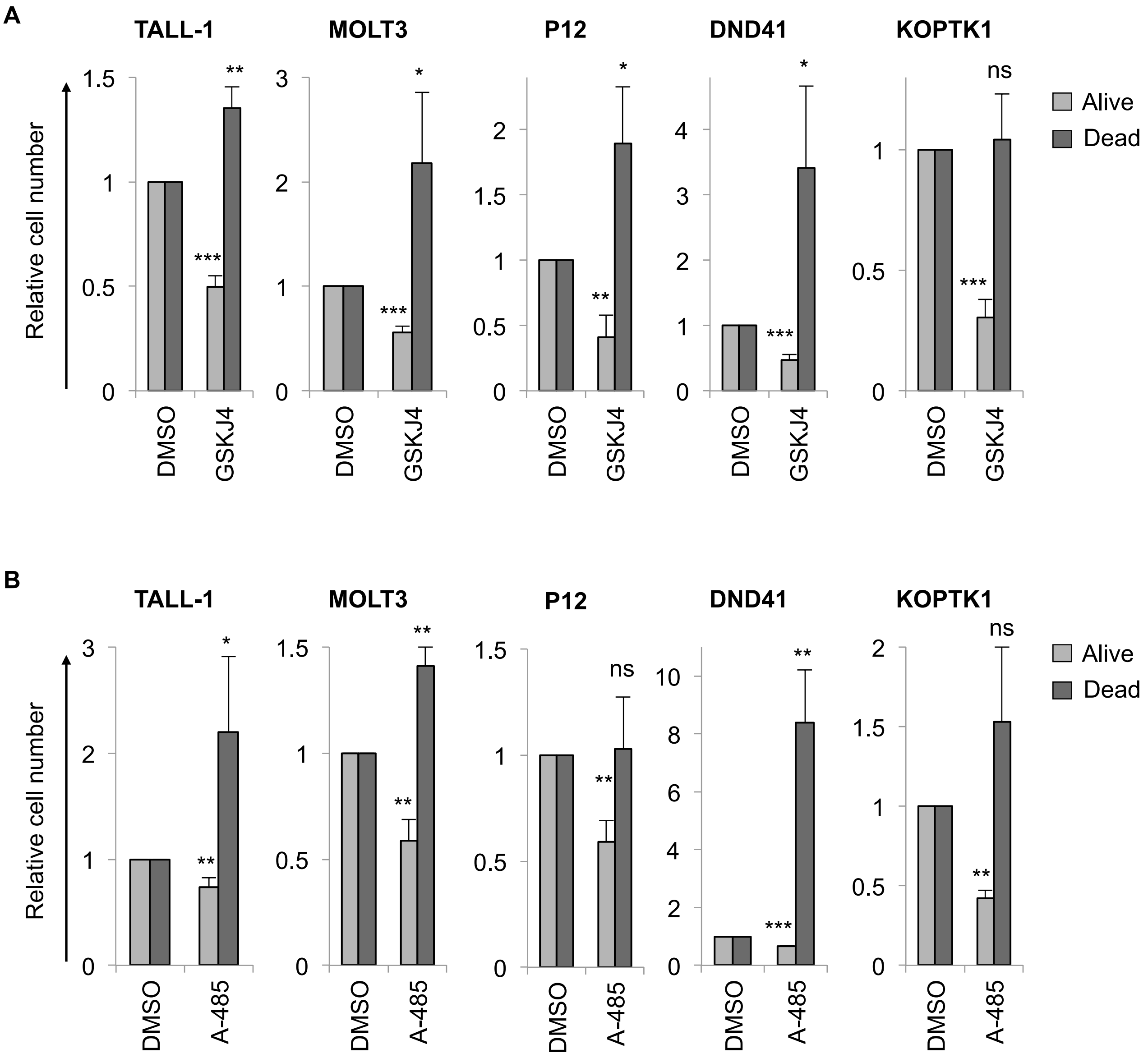

Supplementary Figure 5. Number of viable and dead cells were detected via trypan blue exclusion assay. Relative cell number reported in charts represents the mean of 4 replicates normalized to cell number in DMSO-treated cells ± Standard Deviation of the Mean (S.D,); *$P < 0.05$, **$P < 0.01$, ***$P < 0.001$.

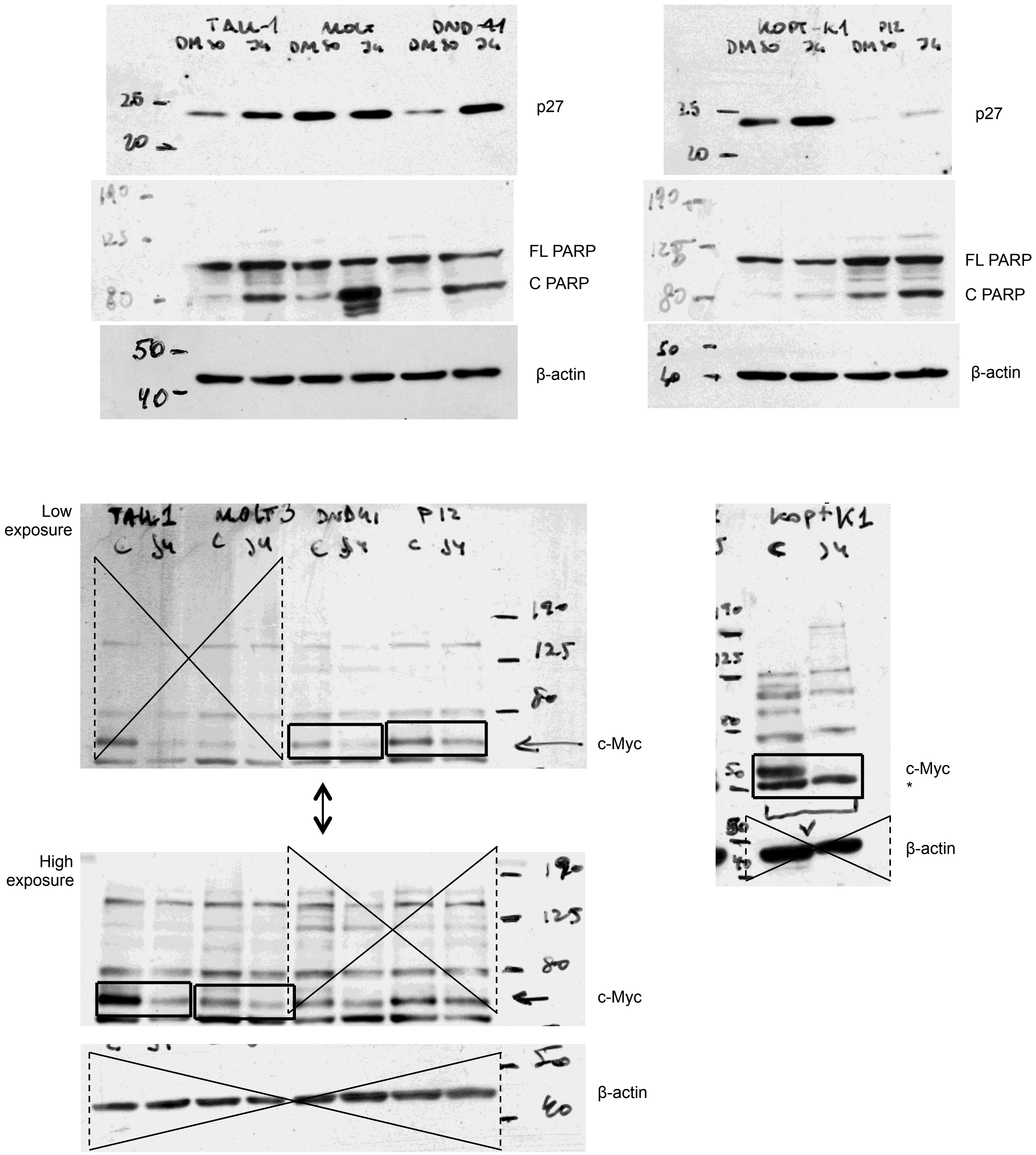

Supplementary Figure 6. Uncropped Western Blots related to main Fig. 5B.

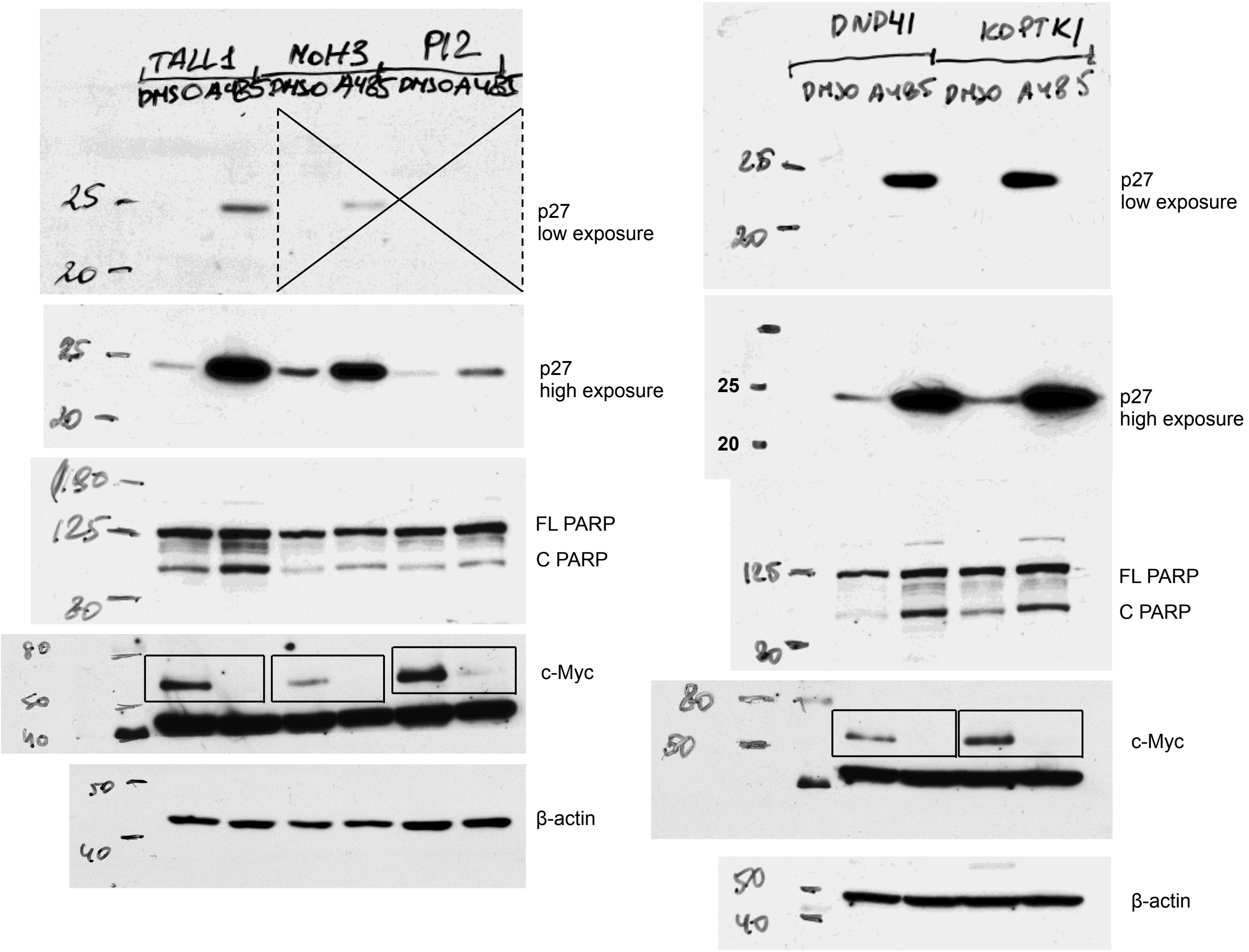

Supplementary Figure 7. Uncropped Western Blots related to main Fig. 5D.

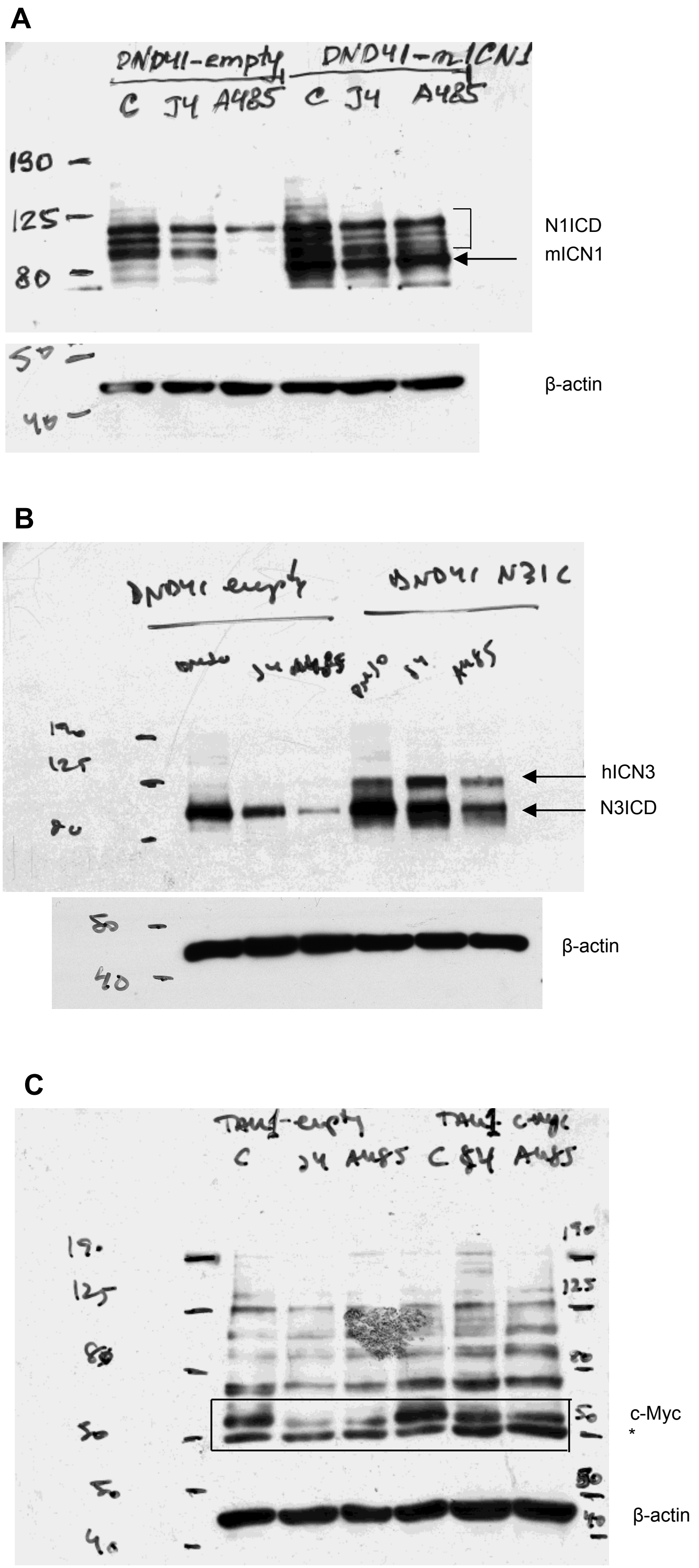

Supplementary Figure 8. Uncropped Western Blots related to main Fig. 6.
